# DISPARITIES IN ACCESSING DIFFERENT TYPES OF LONG-TERM CARE SERVICES IN TAIWAN

**DOI:** 10.1093/geroni/igz038.565

**Published:** 2019-11-08

**Authors:** Ya-Mei Chen, Shih-Chi Lin, Hsiao-Wei Yu, Shwu-Chong Wu, Shih-Cyuan Wu, Ying-Chieh Wang, Tung-Liang Chiang

**Affiliations:** 1 National Taiwan University, Taipei, Taiwan; 2 National Yang-Ming University, Taipei, Taiwan; 3 Chang Gung University of Science and Technology, Taipei, Taiwan

## Abstract

A growing body of evidence documents pervasive social and demographic factors relating to disparities in long-term care (LTC). In 2007, Taiwan implemented its Ten-year Long-Term Care Plan version 1.0 (TLTCP 1.0) that aimed to develop a home-and community-based (HCBS) LTC system. In 2016, Taiwan began to implement TLTCP 2.0. To continue providing effective LTC, this study aimed to assess the disparities in access to LTC services using Taiwan’s LTC claim database from 2010 to 2013. A total of 87,438 older adults who had applied for LTC services from the TLTCP 1.0 were included. The study assessed LTC disparities related to five sociodemographic factors, including age, gender, living status, urbanization, and income status. Sixteen types of LTC services, including HCBS, home-based professional care, meal services, transportation, and institutional services were assessed. After controlling for the level of disability, we found that those who were older-old (age 80> over), male, and low-income were less likely to use HCBS, but more likely to use institutions services (p < 0.001). We also found that those who lived in the city were more likely to use HCBS and transportation services (p < 0.001). Yet, older adults living alone were more likely to use home care and meal services but not other types of LTC services (p < 0.001). In conclusion, the social disparities in access to LTC services in Taiwan remains, suggesting LTC 2.0 should continue monitoring and placing the LTC equity issue on the top priority.

